# Visualization of Extracellular Matrix Components within Sectioned *Salmonella* Biofilms on the Surface of Human Gallstones

**DOI:** 10.1371/journal.pone.0089243

**Published:** 2014-02-14

**Authors:** Joanna M. Marshall, Alan D. Flechtner, Krista M. La Perle, John S. Gunn

**Affiliations:** 1 Department of Microbial Infection and Immunity, Center for Microbial Interface Biology, The Ohio State University, Columbus, Ohio, United States of America; 2 Department of Veterinary Biosciences, Comparative Pathology and Mouse Phenotyping Shared Resource, The Ohio State University, Columbus, Ohio, United States of America; Quuen's University Belfast, United Kingdom

## Abstract

Chronic carriage of *Salmonella* Typhi is mediated primarily through the formation of bacterial biofilms on the surface of cholesterol gallstones. Biofilms, by definition, involve the formation of a bacterial community encased within a protective macromolecular matrix. Previous work has demonstrated the composition of the biofilm matrix to be complex and highly variable in response to altered environmental conditions. Although known to play an important role in bacterial persistence in a variety of contexts, the *Salmonella* biofilm matrix remains largely uncharacterized under physiological conditions. Initial attempts to study matrix components and architecture of the biofilm matrix on gallstone surfaces were hindered by the auto-fluorescence of cholesterol. In this work we describe a method for sectioning and direct visualization of extracellular matrix components of the *Salmonella* biofilm on the surface of human cholesterol gallstones and provide a description of the major matrix components observed therein. Confocal micrographs revealed robust biofilm formation, characterized by abundant but highly heterogeneous expression of polysaccharides such as LPS, Vi and O-antigen capsule. CsgA was not observed in the biofilm matrix and flagellar expression was tightly restricted to the biofilm-cholesterol interface. Images also revealed the presence of preexisting *Enterobacteriaceae* encased within the structure of the gallstone. These results demonstrate the use and feasibility of this method while highlighting the importance of studying the native architecture of the gallstone biofilm. A better understanding of the contribution of individual matrix components to the overall biofilm structure will facilitate the development of more effective and specific methods to disrupt these bacterial communities.

## Introduction

*Salmonella enterica* serovar Typhi (*S*. Typhi) is the causative agent of typhoid fever, a severe systemic illness responsible for over 21 million new infections annually [1]. Although rare in western countries, typhoid fever continues to pose a major threat to public health in regions of Asia, Africa and South America [2]. *S*. Typhi is a highly virulent, human restricted pathogen, which is typically transmitted through the fecal-oral route by ingestion of contaminated food or water. Characterized by high fever and malaise, the illness usually subsides within 2–4 weeks with effective treatment [3]. However, 3–5% of infected patients go on to become chronic asymptomatic carriers [4], [5], continuing to shed viable bacteria in their feces for over a year and serving as a critical reservoir for the continued spread of disease [6]. A primary mechanism of chronic *S*. Typhi carriage is believed to be formation of bacterial biofilms on the surface of cholesterol gallstones in the gallbladder [7]. Unfortunately, there remains no effective non-surgical treatment to resolve chronic *S*. Typhi carriage in the presence of gallstones [8], [9].

Biofilm formation is a widely conserved mechanism of bacterial persistence characterized by adherence to a surface and subsequent formation of a protective extracellular matrix comprising up to 90% of the biofilm mass [10]. Biofilm matrices are complex aggregations of hydrated macromolecules typically composed of a combination of extracellular polymeric substances (EPS) such as proteins, lipids, polysaccharides and nucleic acids [10], [11]. Encased within the biofilm matrix, bacteria become highly resistant to mechanical disruption, clearance by the host immune system and many otherwise effective antimicrobial therapies [12]. Resolution of biofilm-mediated chronic infections is notoriously difficult and poses a significant problem to the medical community. Recent attempts at development of therapeutics to resolve recalcitrant biofilm-mediated infections by organisms such as *Staphylococcus*, *Pseudomonas*, *Haemophilus, Escherichia* and *Bacillus* have focused on targeting key structural components of the biofilm extracellular matrix (ECM) [13]–[16]. Although this strategy has shown promise in other organisms, identification of similar targets in *Salmonella* biofilms will require in-depth study of the biofilm matrix.

The composition of the *Salmonella* spp. biofilm matrix has been demonstrated to be highly variable depending upon the environment and substratum upon which it is formed [17]–[19]. Proteinaceous factors proposed to be involved in *Salmonella* multicellular behavior include flagella, BapA, OmpC and curli fimbriae (composed of polymerized CsgA) [20]–[22]. Matrix polysaccharides may include O-ag capsule or Vi-ag capsule, LPS, colanic acid and cellulose [19], [22]–[26]. Although previous work from our lab and others have found variable roles for O-ag and Vi-ag capsules in multicellular behavior [26]–[28], these protective surface polysaccharides are closely associated with the outer membrane of *S*. Typhimurium and *S*. Typhi respectively, making them a likely component of the biofilm matrix.

Previous attempts to study the ECM in salmonellae have characterized overall biomass or apparent biofilm quality using mutants deficient in specific ECM components or following enzymatic treatment [19], [28], [29]. These studies have greatly furthered our understanding of the composition and function of the biofilm matrix, but have also revealed it to be a complex and highly variable structure. Possible drawbacks to these methods of examining the overall biomass include lack of enzymatic or quantitative specificity and potential compensatory production of alternative ECM components. Furthermore, studies of the ECM in nontyphoidal salmonellae are frequently conducted at low temperatures (<28°C) and osmolarity. Although such experimental conditions are surely relevant to studies of environmental surface adherence and persistence, production of important ECM components such as cellulose and curli is decreased at 37°C [30]–[32], Furthermore, previous work from our lab has demonstrated that bacterial gene expression is alternately regulated by growth on cholesterol substrates and in response to bile [6], [33], [34]. Biofilms forming on cholesterol gallstones in the bile-rich gallbladder are exposed to a unique environment. Collectively, previous data imply that the macromolecular components involved in the *Salmonella* gallstone biofilm ECM likely differ from those ECM components reported to be critical for biofilm formation in other environments.

Understanding the relative abundance and organization of individual biofilm matrix components will facilitate progress toward the goal of developing targeted therapies to disrupt the biofilm structure. In this work we sought to further characterize the *Salmonella* gallstone biofilm by sectioning and specifically labeling molecules proposed to be important components of the ECM within the native structure of biofilms grown on the surface of patient gallstones at 37°C. We have optimized the sectioning procedure and demonstrated its potential for use in both human and animal studies through preliminary analysis of *S*. Typhi and *S*. Typhimurium biofilm composition and architecture. Our results indicate that the biofilm matrix is largely composed of polysaccharides under the conditions tested and that proteins and nucleic acids represent relatively minimal contributors to the overall ECM biomass. This work complements previous studies and highlights the importance of understanding the organization of ECM constituents within the architecture of the biofilm matrix.

## Results

*Salmonella* Typhi are able to persist asymptomatically in the gallbladder of human hosts through the formation of biofilms on the surface of cholesterol gallstones. Previously, our group and others have reported the presence of matrix-encased bacterial communities associated with the surface of gallstones [7], [28], [35], [36]. The extracellular matrix is a complex and variable structure conferring increased antimicrobial resistance and aiding bacterial aggregation. Although therapies aimed at resolving biofilm-mediated infections by targeting specific ECM components have demonstrated promise in other organisms, our understanding of the *Salmonella* biofilm ECM *in vivo* is limited. The method described here permits visualization and specific labeling of preserved bacterial biofilms grown *ex vivo* on the surface of patient cholesterol gallstones. Visual analysis of the molecular components within the biofilm ECM was the primary goal of this study, and to accomplish this, we developed a new histologic slide preparation permitting visualization of human gallstones with *Salmonella* biofilms on their surface.

### Gallstone sectioning procedure

**Figure 1:**
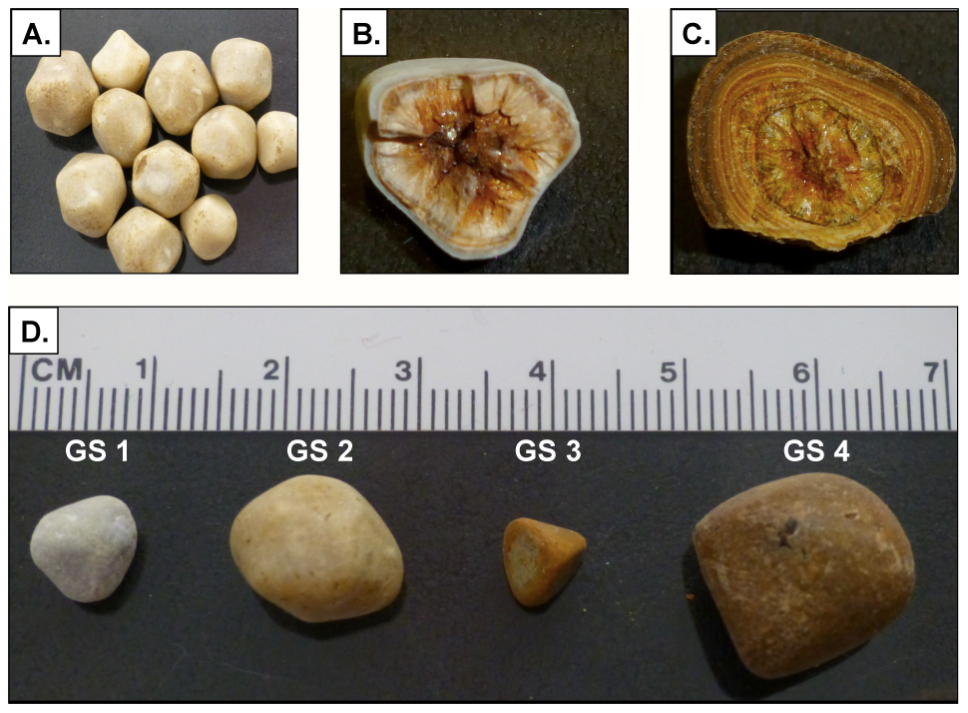
Gross morphological features of patient gallstones employed in this study. **A**) Representative image of a group of gallstones retrieved from a single patient (GS 2) demonstrating consistent size and visual appearance. **B**) Cross section of gallstone (GS 1) and **C**) Cross section of gallstone (GS 4), revealing heterogeneous appearance throughout a single gallstone. **D**) The sectioning procedure was attempted on 4 patient gallstones of varying size and visual appearance. Gallstone sample GS 1 was selected for use in biofilm analysis experiments based on the success of the sectioning technique and having characteristics consistent with gallstones containing a high percentage of cholesterol.

Gallstones are categorized broadly as either cholesterol or pigment, based on having either cholesterol or calcium billirubinate as their primary constituent [37]. Gallstone type and nomenclature is based upon macroscopic characteristics, which have been shown to have a high correlation with gallstone composition (>94%) [38]–[40]. By definition, cholesterol gallstones contain over 50% cholesterol but frequently have significant amounts of calcium billirubinate as a secondary constituent, which may give the stone a brown appearance [41], [42]. All gallstone samples employed in this study were categorized as cholesterol gallstones, having previously been analyzed by infrared spectroscopy [43] and determined to be composed of >50% cholesterol with a mean calcium billirubinate content of 35%. Patient gallstone samples contained multiple gallstones collected from a single individual which were similar in size and appearance to other stones from the same patient (**Fig. 1A**). Analysis of bisected gallstones revealed heterogeneous composition within individual gallstones, reflecting the complex process of lithogenesis (**Fig. 1B–1C**) [44]. Gallstones of varying size and appearance were selected from 4 individual patients (**Fig. 1D**) for initial testing of the sectioning procedure. Sectioning was also attempted on 3 murine gallstones retrieved from laboratory animals fed a high-cholesterol diet for 10 weeks {Gonzalez-Escobedo, 2013 #1305}. A number of unsuccessful methods for processing and sectioning were attempted which included directly bisecting the gallstones, cryosectioning, and omission of overnight processing and/or decalcification, all of which resulted in gallstone dissolution during subsequent processing steps. Optimal results were achieved using immersion fixation and mild formic acid/sodium citrate decalcification followed by overnight processing, paraffin embedding and sectioning. White (cholesterol) gallstones responded well to the processing method while brown-pigmented gallstones (containing an increased proportion of calcium bilirubinate) [45], [46] did not withstand the decalcification procedure. Murine gallstones shattered during sectioning due to their brittle nature however efforts are ongoing to provide additional reinforcement by embedding murine gallstones in resin prior to sectioning.

### Analysis of *S*. Typhi and *S*. Typhimurium biofilms grown on the surface of Gallstones from patient 1 (GS 1)

**Figure 2:**
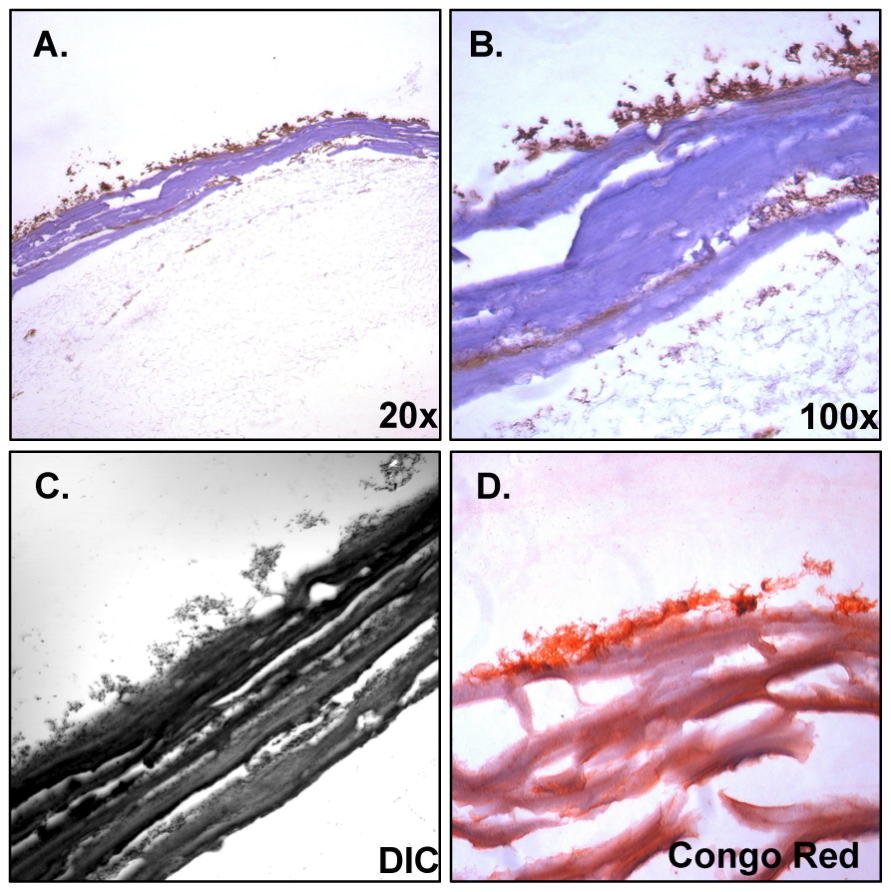
Photomicrographs of sectioned human gallstones with surface associated biofilms of *Salmonella* Typhimurium. Gallstone surface (pale purple band) with associated biofilm stained with anti-*Salmonella* polyclonal antibody (brown) visualized at **A**) 20×**B**) 100× magnification and **C**) Differential interference contrast (DIC) micrographs reveal well-preserved surface-associated bacterial communities. **D**) Congo Red staining indicates abundance of β-amyloid fibrils and/or acidic polysaccharides.

**Figure 3:**
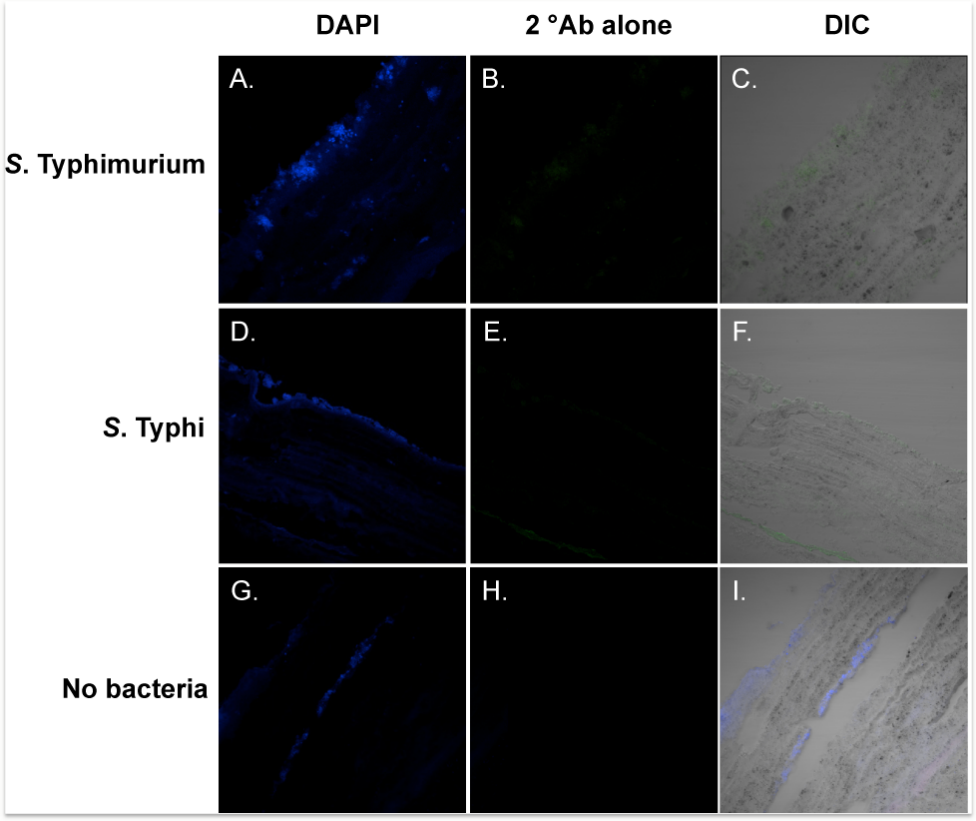
IF staining controls demonstrating minimal non-specific fluorescence. Sectioned patient gallstones with **A–C**) *S*. Typhimurium biofilm, **D–F**) *S*. Typhi biofilm or **G–I**) no bacteria control. All sections were stained with relevant primary and secondary antibodies and counterstained with DAPI. Micrographs of control gallstones (**G–I**) incubated in sterile media, exhibit regions of DAPI staining resembling pre-existing bacterial aggregates within the gallstone.

Gallstones from patient 1 (**Fig. 1D** **, GS 1**) withstood the sectioning procedure very well and exhibited gross characteristics consistent with that of pure cholesterol gallstones [47]. Additionally, patient sample GS 1 contained >20 gallstones of highly similar size and appearance and was therefore selected for use in biofilm sectioning and visualization experiments. Bacterial biofilms were grown on gallstone surfaces by incubating gallstones GS 1 in culture for 6-days prior to processing. Microscopic analysis of H&E stained sections labeled for *Salmonella* revealed that bacterial biofilms remained attached to the gallstone surface throughout processing (**Fig. 2A–D**). The best-preserved portions of the biofilm appeared to be associated with crevices and indentations in the stone surface. This may be an artifact of the rotation of the culture tubes to facilitate aeration or could reflect a preference for the increased surface contact provided by these topographical features. Immunofluorescent (IF) staining controls (**Fig. 3**) revealed minimal, low-level background fluorescence, likely due to nonspecific staining as well as background autofluorescence from cholesterol and formalin fixation (**Fig. 3B, 3E, 3H**) but this was nominal as compared to reactivity of specifically labeled components. Examination of control gallstones incubated in sterile LB alone and similarly stained for surface antigens, revealed the presence of pre-existing bacterial aggregates both on the surface and several layers into the interior of the stone (**Fig. 3G–3I**). Previous research has linked biliary infection with gallstone development and indicated that bacteria may act as the nucleating factor initiating the formation of both pigment and cholesterol gallstones. [40], [47], [48]. In this study, although detectable bacteria appeared encased within the gallstone structure, none was observed at the center of the gallstone as would be expected of an object that had acted as a nucleating factor for initial cholesterol precipitation. However, bacteria may have participated in nucleation but were not detected by our antibodies. The suspected layer of bacteria in the gallstone was unreactive with monoclonal *S*. Typhi and *S*. Typhimurium antibodies (**Fig. 3H**) but strong reactivity was observed with polyclonal anti-*Salmonella* antibody (**Fig. 2A–B**) indicating other salmonellae or *Enterobacteriaceae*.

### Analysis of biofilm extracellular matrix components

**Figure 4:**
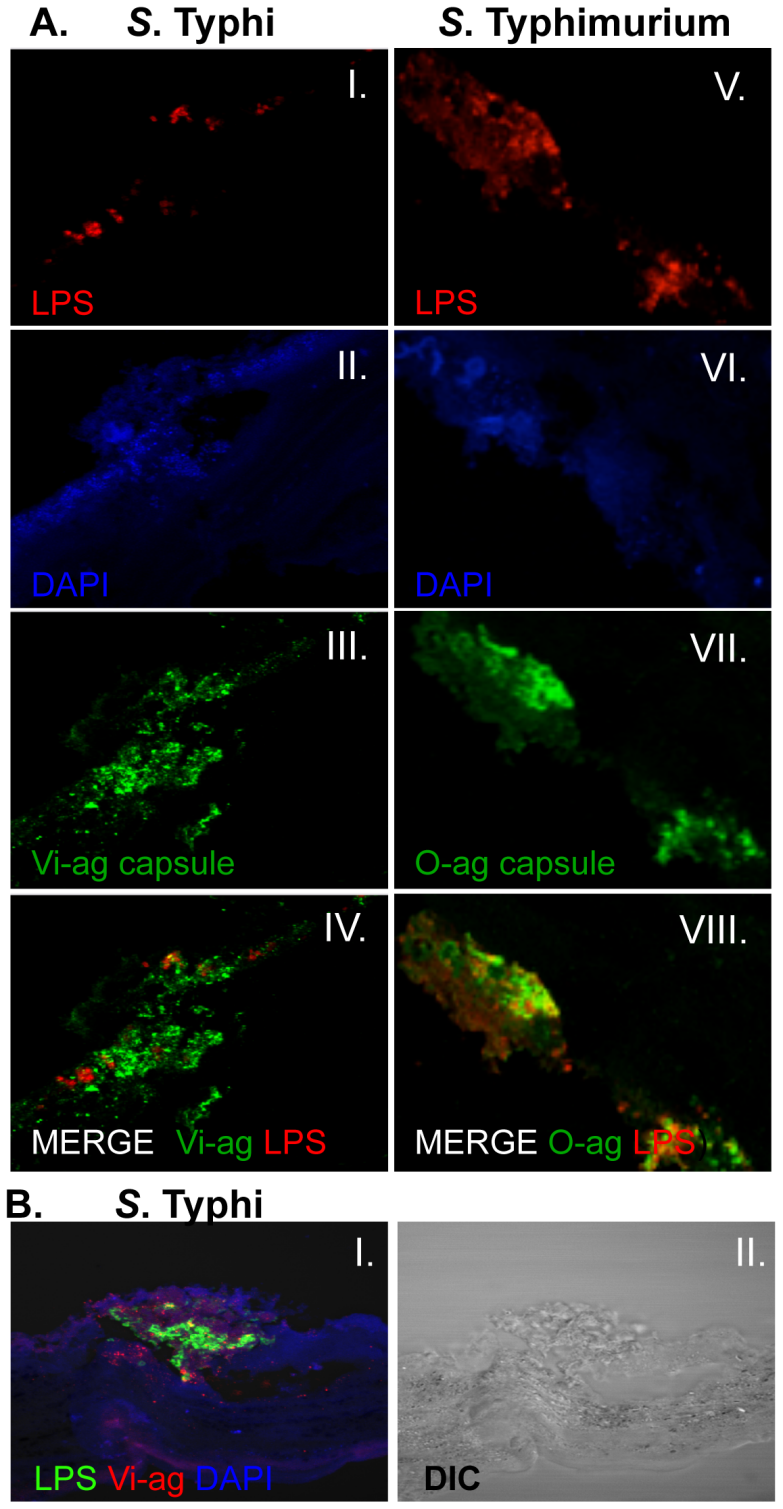
Visualization of polysaccharide antigens in DAPI counterstained *Salmonella* biofilms grown on human gallstones. *Upper panel* **3A**) Biofilms of *S*. Typhi (**I–IV**) and *S*. Typhimurium (**V–VIII**) grown in LB without bile. Sections were stained for LPS (red: **I, V**) Vi-ag (green, **III**) or O-ag capsule (green, **VII**). Merged images of LPS and Vi-ag/O-ag capsule (**IV, VIII**) illustrate the heterogeneous detection of these polysaccharides within the biofilm. *Lower panel* **3B**) Representative image of *S*. Typhi biofilm section grown in LB with 3% bile, counterstained with DAPI and fluorescently labeled for LPS (green) and Vi-antigen (red). Confocal (**I**) and DIC (**II**) images demonstrate the intimate association of the biofilm with gallstone surfaces and the abundance of polysaccharides in the extracellular matrix.

Sectioning and labeling of *ex vivo* biofilms revealed that polysaccharide and proteinaceous components could be readily detected and visualized in the *Salmonella* gallstone biofilm matrix (**Fig. 4**). LPS (**Fig. 4A–I, 4A–V, 4B–I**) and capsular polysaccharides (**Fig. 4A–III, 4A–VII, 4B–I**) were clearly visible within the biofilm structures of both *S*. Typhi and *S*. Typhimurium. Interestingly, within the biofilm, heterogeneous detection of both LPS and capsule was observed, with many DAPI-positive cells staining for capsule alone, LPS alone or both (**Fig. 4A–IV, 4A–VIII, 4B–I**). While Vi-ag was readily detected within the *S*. Typhi biofilm, LPS staining occurred on fewer than 20% of the cells staining with DAPI (**Fig. 4A–IV, 4B–I**). Similar results were observed in free bacterial microcolonies and biofilm sections stained for LPS or capsule alone (data not shown).

**Figure 5:**
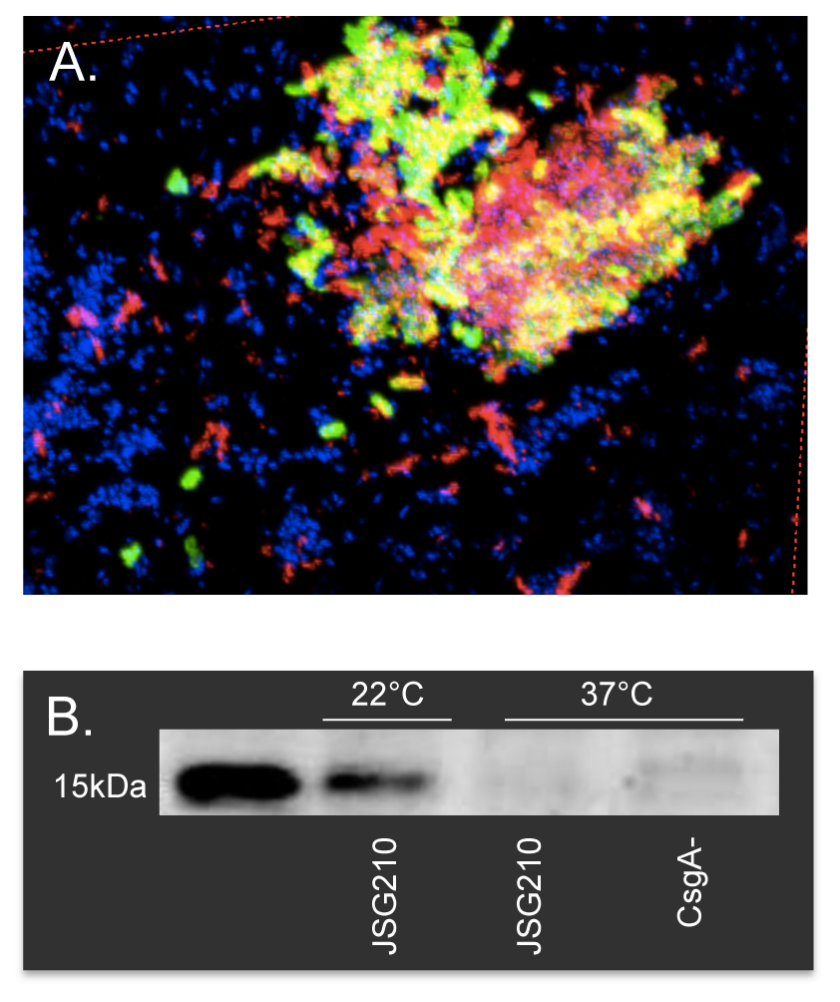
Flagella and CsgA detection in bacterial microcolonies and whole cell lysates. **A**) Although minimally detected in the gallstone biofilm extracellular matrix, confocal scanning micrographs demonstrate that flagella (red) and CsgA (green) are readily detectable in bacterial microcolonies grown in matrix inducing conditions (LBNS, 22°C, 5days). **B**) Western blot detecting CsgA (≈15 kDa) in whole cell lysates of *S*. Typhimurium wild type JSG210 or *δcsgA* reveal CsgA production at 22°C but not at 37°C.

**Figure 6:**
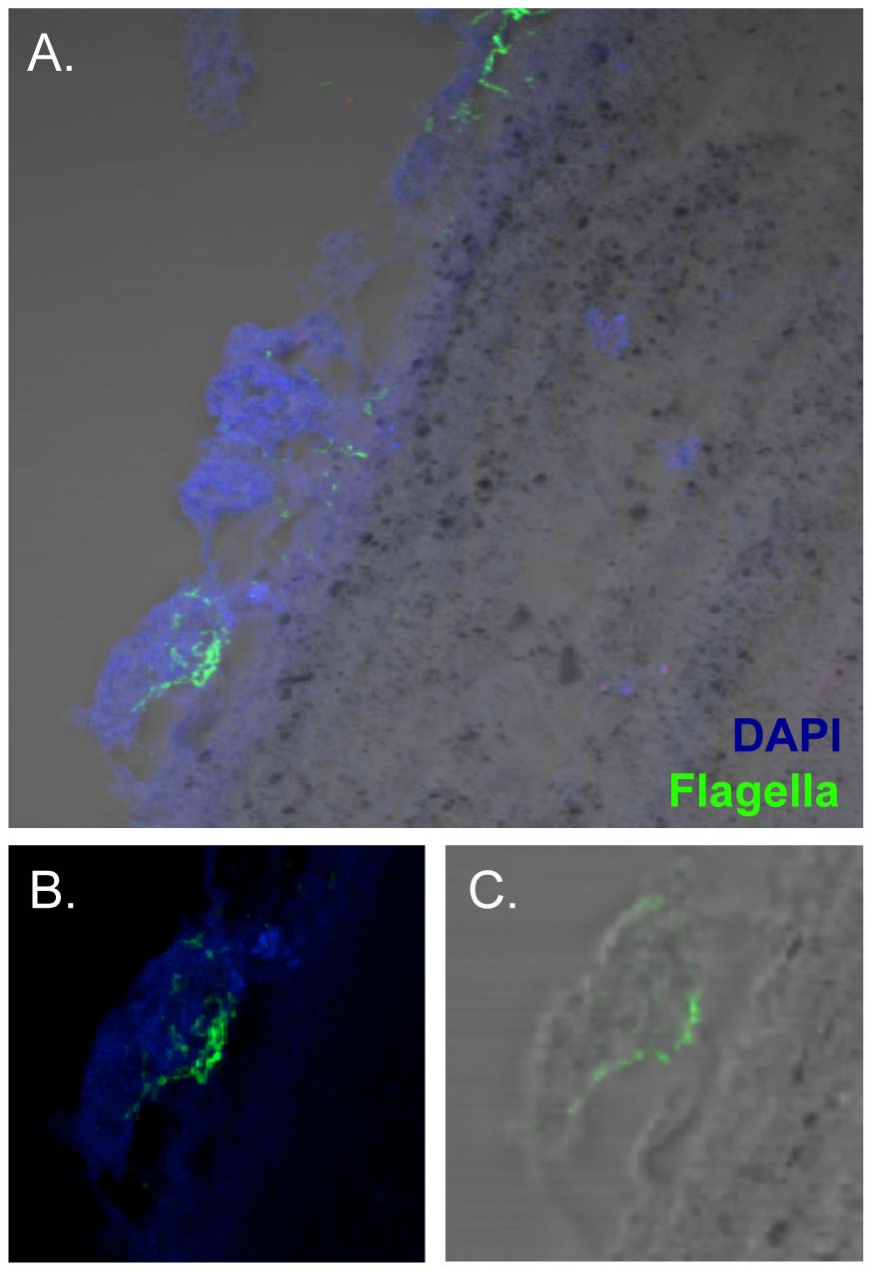
Visualization of flagella in DAPI counterstained gallstone biofilm sections. **A**) Confocal micrograph of *S*. Typhimurium biofilm demonstrating localization of flagella. Magnified CSLM (**B**) and DIC/CSLM merge (**C**) indicate that flagella is restricted to the interface of the biofilm and cholesterol gallstone surface.

Although previous reports have indicated proteinaceous components such as curli fimbriae (CsgA) and flagella to be important constituents of the biofilm matrix, neither molecule was abundantly detected within the gallstone biofilm grown at the physiologically relevant temperature of 37°C (data not shown). To ensure that IF staining techniques employed were suitable for detection of ECM components within densely packed bacterial communities, free bacterial microcolonies grown in matrix-inducing conditions (LBNS, 22°C ×5 d) displaying dry and rough morphology [32], were fixed and stained, demonstrating that when present, these putative matrix components could be specifically labeled and visualized within the microbial community (**Fig. 5A**). Western blotting of whole cell lysates revealed that CsgA was undetectable in cells grown at 37°C (**Fig. 5B**). Flagella, although minimally expressed, were tightly localized to the surface of the gallstone at the interface between the biofilm and cholesterol surface (**Fig. 6**). This observation is consistent with previous findings implicating flagellar filaments in cholesterol attachment [20].

## Discussion

Formation of cholesterol gallstones is a complex and slow process, with composition differences from patient to patient [49]. The methods described herein for histologic preparation were most successful on cholesterol gallstones and met with less success on pigmented calcium bilirubinate gallstones. Photomicrographs reveal the presence of what appear to be bacterial aggregates in the sub-surface layers of the stone, although no organisms were cultured from negative control gallstones incubated in media alone. Previous papers have reported a potential role for bacterial infection during the formation and nucleation of gallstones [50], [51]; however, no bacteria detectable with our antisera were observed in the central core of patient samples observed in this study. These sub-surface organisms stained with DAPI and reacted strongly with a polyclonal anti-*Salmonella* antibody, but did not react with monoclonal *S*. Typhi or *S*. Typhimurium antibodies. Previous studies have reported that numerous bacterial species associate with gallstones, and bacteria or bacterial DNA is detectible in the majority of patient gallstone samples (<87%) [47], [52]. The observations made in this study are consistent with these previous reports indicating that bacteria are frequently detected in association with patient gallstone samples. Our data imply that the patient from which gallstones GS 1 were retrieved may have experienced a previous gallbladder infection with another *Salmonella* serovar or another *Enterobacteriaceae*, during which bacteria attached to and became encased within the forming stone. Although further clinical details for the patient from which gallstones GS 1 were retrieved are not available, the majority of patients undergoing cholecystectomy do so as a result of acute cholecystitis, which has been reported to be co-incident with bacterial biliary infections in 22–58% of cases [53]. Therefore it is possible that bacterial infection occurred shortly before gallbladder removal and may have been the precipitating event leading to symptomatic gallbladder disease and subsequent cholecystectomy.

Biofilm formation on gallstone surfaces appeared to be more robust at the site of imperfections and indentations in the ‘crust’ of the stone and, in agreement with previous findings, was more robust in the presence of bile [28]. Although chronic gallbladder carriage of *Salmonella* in humans has been reported primarily to be a complication of infection with *S*. Typhi, long-term colonization has been reported with nontyphoidal salmonellae as well [54]–[57] and current murine models of chronic gallbladder colonization employ *S*. Typhimurium. This method proved equally suitable for analysis of gallstone biofilms by either *S*. Typhi or *S*. Typhimurium, making it likely to be of use in future animal studies. The sectioning procedure outlined herein was tested on several gallstone types with success on most; however, this preliminary study to analyze biofilm ECM components on the gallstone surface was conducted with gallstones from a single patient (GS 1). This gallstone sample was selected based on its amenability to the sectioning procedure, its visual similarity to previously described pure cholesterol gallstones and the high number of gallstones available from this patient for experimental replication and controls.

Bacterial components directly labeled and observed in this study included LPS, O-ag capsule, Vi-ag capsule, DNA and flagella, several of which appeared to be spatially restricted or heterogeneously expressed within the biofilm. Flagella, although a relatively minor component of the total matrix, appeared tightly localized to the biofilm-cholesterol interface, supporting the previous observation indicating that flagellar filaments mediate bacterial binding to cholesterol surfaces [20]. Conversely, Congo red, a dye that binds cellulose, acidic polysaccharides and the CsgA β-amyloid fibrils of curli, stained the biofilm structure extensively, possibly indicating the presence of additional unlabeled structures such as colanic acid or curli fimbriae. Sections were stained with α-CsgA to determine if Congo red staining was due to abundant curli fimbriae in the biofilm, but no CsgA was detected. Previous results have indicated CsgA to be an important component in *Salmonella* biofilm formation and surface adherence [22], but expression is maximal at ambient temperatures (≤28°C) [58]. Staining of free bacterial microcolonies grown at 22°C demonstrated that when present, CsgA is easily detected. Additionally, western blotting of whole cell lysates revealed that CsgA is not produced at 37°C, indicating that the absence of CsgA reflects an absence of the protein in the ECM and not an artifact of staining. These results led to the conclusion that colanic acid or enterobacterial common antigen is more likely to be the source of Congo red staining, thereby illustrating the changing nature of the biofilm matrix in response to environmental conditions and highlighting the fact that matrix components may play a structural role that is disproportionate to their relative abundance.

Capsular polysaccharides O-ag and Vi-ag capsule were abundant in biofilms of *S*. Typhimurium and *S*. Typhi, respectively. In spite of its apparent abundance, Vi-ag capsule has been shown not to be important for structural integrity of *S*. Typhi biofilms [28]. Conversely O-ag capsule, which appeared to be less abundant in *S*. Typhimurium biofilms than Vi-ag capsule in *S*. Typhi, has been reported to be important for multicellular behavior in *S*. Typhimurium [26] but not for *S*. Enteritidis [27]. In *S*. Typhi biofilms, fewer than 20% of cells that stained for Vi-ag capsule and DAPI were labeled for LPS. While this could represent an artifact of staining, similar staining of free bacterial microcolonies has also demonstrated that LPS and capsule are not equally detectible on all cells within the bacterial community. DAPI counterstains were employed to indicate bacterial nucleic acid, but also appeared to demonstrate diffuse regions of staining on the surface of *S*. Typhi biofilms grown in the presence of bile. Although this may indicate the presence of extracellular DNA in the biofilm matrix, further studies will be needed as we did not employ a staining mechanism that permits differentiation of intracellular and extracellular DNA.

Although nucleic acids and proteinaceous components are likely to play an important role in biofilm structure, the results of this work indicate that polysaccharides comprise the large majority of the *Salmonella* biofilm matrix. It is interesting to note that structurally important components such as flagella represent a small fraction of the overall biomass as compared to molecules such as Vi-ag, which are abundant in spite of playing a seemingly minor structural role. *Salmonella* Pathogenicity Island 7 (SPI7) encodes the genes responsible for Vi-ag capsular assembly. SPI7 is absent in *S*. Typhimurium; therefore Vi-ag capsule is not produced by this serovar. Although it is possible that Vi-ag is simply trapped within the forming biofilm matrix, it is tempting to speculate that the overabundance of this energetically expensive carbohydrate structure implies an alternative non-structural role. In fact, Vi-ag has been demonstrated to be a key stealth component of *S*. Typhi during the acute phase of disease, allowing it to avoid innate immune detection in the gut and proceed to systemic dissemination [59]. It is possible that proteinaceous components, even in relatively low abundance, provide structural integrity, while the polysaccharide components function in a chemically and/or immune-protective manner, or that structural integrity requires complex interactions between both polysaccharide and proteinaceous components as has been observed in *Pseudomonas aeruginosa* [60]. Further studies will focus on refining this technique for use in animal models and analyzing the response of biofilm to antimicrobial therapies.

The preliminary structural observations of *Salmonella* gallstone biofilms described herein raise several interesting questions with potential implications in disease and therapeutics. Viable bacteria encased in patient gallstones have been previously reported [61] and microscopically observed [62]. Clinically, this scenario could greatly increase resistance to clearance and duration of persistence within the host and would almost certainly hinder the reliability of culture and immune-based diagnostics. Furthermore, the ability of organisms within the biofilm community to limit the surface expression of highly immunogenic structures such as LPS and flagella, has implications both for generation of protective immunity as well as immune-based detection and resolution of *Salmonella* gallstone biofilms. Although further studies are needed to determine how broadly applicable these findings are, we anticipate that this method will be useful for future work aimed at characterizing the biofilm architecture and structural components both during the disease state and in response to the choice of treatment.

## Materials and Methods

### Bacterial strains and growth conditions

Wild-type reference strains for *S*. Typhi Ty2 (gift of Dr. Renato Morona, University of Adelaide) or *S*. Typhimurium 14028S (ATCC) were struck on LB agar, cultures were inoculated from an isolated colony and grown overnight in LB liquid medium with aeration at 37°C. For matrix inducing conditions used in western blotting and microcolony staining of CsgA/FliC, cells were grown on LB no salt agar for 5 days at 22°C.

### Ethics Statement

This project did not involve the use of animals or interaction with human subjects. Laboratory experiments involved only existing, previously collected pathological specimens obtained from Dr. Wayne Schwesinger [38], Department of General Surgery, University of Texas Health Science Center at San Antonio, San Antonio, Texas. Specimens were not collected specifically for the current research and were anonymously analyzed having been assigned de-identified numbers, which could not be traced back to the patients and with no protected health information available to the researcher. There is no link between patient identifiers and the data. As such, this research does not meet the definition of “research involving human subjects” per the Ohio State University Human Research Protection Program Policies and Procedures, IRB Policy Committee revision 02/02/13 and is not subject to approval or exemption.

### Patient Gallstones

Gallstones employed in this study were previously collected and characterized in a clinical study of gallstone composition and epidemiology [38]. Infrared spectroscopy of powdered gallstones was employed to determine gallstone composition and all gallstones used in this study were at least 50% cholesterol with an average composition of 52% cholesterol and 35% calcium billirubinate. The sectioning procedure was initially tested on 4 patient gallstones: one large and one small gallstone of either white or brown appearance. Gallstones from patient 1 (GS 1) exhibited the characteristic appearance of pure cholesterol gallstones [47], responded well to the sectioning procedure and came from a patient sample with a large number of highly similar gallstones Therefore this gallstone was employed for use in subsequent biofilm analysis experiments.

### Biofilm formation

Patient gallstones were placed in 15 mL borosilicate glass tubes using sterile forceps. To determine pre-existence of viable organisms, gallstones were incubated overnight in 5 mL of sterile LB, which was subsequently plated on LB and XLD agar. For biofilm cultivation, overnight bacterial cultures were normalized to OD_600_ 0.8, diluted 1∶100 (≈2×10^6^ CFU/ml) and inoculated into LB +/−3% ox bile. Biofilm cultures were maintained at 37°C with aeration and media was replaced every 24 hours for 6 days to allow biofilm growth. On days 2 and 6, planktonic culture removed prior to washing was enumerated by plating on LB and XLD agar. Gallstone biofilm analysis experiments were repeated twice by conducting 2 separate biofilm growth experiments on separate gallstones from the same patient. Immunofluorescent (IF) labeling experiments were repeated a minimum of 2 times. All reported observations were consistent among biological and technical replicates.

### Western blotting

The CsgA monomeric subunit of curli fimbriae was detected in whole cell lysates using a polyclonal antisera generated against *E. coli* CsgA, (gift of Dr. Matthew Chapman, University of Michigan). Western blots were conducted as previously described [63]. In brief, cultures were grown as outlined above, cells were scraped from plates or pelleted from culture media, resuspended in 1 mL 1×PBS pH 7.4 and normalized to A_600_ 0.8. Cells were pelleted again, and the supernatant was removed and pellets were resuspended in 80 µL of 88% formic acid to depolymerize CsgA. Samples were dried using a Speed-Vac and resuspended in 35 µL H_2_O. Lamelli loading buffer was added to a final volume of 70 µL and 7.5 µL was loaded onto a 15% SDS-PAGE gel along with Biorad Western C chemiluminescent molecular weight standard. Proteins were electrophoresed at 60 V until the dye front reached the bottom of the gel. Proteins in the gel were transferred for 1 h at 60 V to MeOH-activated PVDF membrane and blocked overnight in 5% BSA in TBS. The membrane was incubated with Anti CsgA antibody (1∶10,000 in 5% BSA/TBST for 2 h, 22°C) washed in TBST (3×15 min) and incubated with HRP conjugated goat α-rabbit (1∶12,500 in 5% BSA/TBST for 2 h, 22°C). The membrane was washed in TBST (3×15 min) and visualized using the Bio-Rad ChemiDoc system.

### Gallstone preparation, fixation, embedding and sectioning

Gallstones were prepared by the Comparative Pathology and Mouse Phenotyping Shared Resource at the Ohio State University. Following 6 days of biofilm growth, media was removed and plated for bacterial enumeration as described above. Gallstones were washed twice in sterile PBS prior to immersion fixation in 10% neutral buffered formalin (NBF) for 24 hours at room temperature. Although several alternate methods were attempted (as discussed in results section) optimal results were achieved with the protocol outlined herein. Fixed samples were rinsed in running tap water for 30 minutes and transferred into a 1∶1 solution of 50% aqueous formic acid and 20% aqueous sodium citrate overnight (approximately 16 hours). After gently rinsing in running tap water for 30 minutes, gallstones were transferred back into 10% NBF for standard overnight tissue processing in a Tissue Tek V.I.P. 3000 vacuum infiltration processor (Sakura Finetek USA, Inc., Torrance, CA). In brief, samples were incubated in 10% NBF (2×30 min), 70% ethanol (1×2 hrs), 95% ethanol (3×60 min), 100% ethanol (1×60 min, 1×90 min), toluene (1×60 min, 1×90 min) and submerged in paraffin (3×60 min) prior to carefully embedding in fresh molten paraffin (Paraplast Plus, Sigma-Aldrich, St. Louis, MO) and then allowed to cool. Four micron sections were obtained using a RM2255 automated microtome (Leica Biosystems, Buffalo Grove, IL). Tissue sections were floated in a 48°C water bath and placed on Surgipath Superior Adhesive Slides (Leica Biosystems). The slides were air dried overnight and then transferred into a 60°C oven for 30 minutes. Slides were deparaffinized and hydrated to distilled water by washing in xylene (3×5 min), 100% ethanol (2×1 min), and 95% ethanol (2×1 min), and then briefly rinsed in and transferred to distilled water for IF or immunohistochemical staining.

### Immunohistochemical staining

Slides were treated with DakoCytomation Target Retrieval Solution (Dako, Carpinteria, CA) in a Decloaking Chamber (Biocare Medical, Concord, CA) heated to 125°C and then cooled to 90°C for 10 sec, before cooling with the lid removed for 10 min to unmask epitopes for *Salmonella* detection. Slides were then transferred to a Dako Universal Training Center automatic immunostainer for all subsequent steps at RT. Endogenous peroxidase was inhibited in 3% H_2_O_2_ for 5 min, followed by serum-free protein block (Dako) for 10 min. Sections were incubated with rabbit polyclonal anti-*Salmonella* antibody (1∶100) for 30 min (Novus Biologicals), followed by a biotinylated anti-rabbit secondary antibody (1∶200, Vector Laboratories, Burlingame, CA) for 30 min, and lastly avidin-biotin complex (Vector Laboratories) for 30 min. Signal was developed with 3,3′ diaminobenzidine tetrahydrochloride, counterstained with hematoxylin, and coverslipped prior to viewing with a light microscope.

### Immunofluorescent (IF) staining

Deparaffinized slides were blocked in sterile filtered 5% bovine serum albumin (BSA) in Tris-buffered saline (TBS) pH 7.4 overnight at 4°C. All antibodies were diluted in 5% BSA/TBS+ Tween 20 (TBST). Secondary antibodies were goat or donkey anti-rabbit or mouse. Staining controls included gallstones with no bacteria stained with primary and secondary antibodies and gallstones with bacterial biofilms unstained or stained with either primary antibodies or secondary antibodies alone. 4′,6-diamidino-2-phenylindole (DAPI) was used as a counterstain to permit visualization of DNA. Following antibody incubations (**Table 1**), slides were washed in TBST, rinsed briefly in sterile ddH_2_O and mounted using ProLong Gold anti-fade mounting media. Slides were allowed to dry in the dark at room temperature overnight prior to viewing on an Olympus FV1000 spectral confocal scanning laser microscope. Light images were captured using differential interference contrast (DIC) optic settings.

**Table 1:** Antibodies/stains employed in immunohistochemistry and immunofluorescence slide staining.

Host Species/stain	Target	Dilution	Time, Temperature	Source
Mouse	*S*. Typhi monoclonal α-LPS C01362M	1∶200	1 h, 22°C	Biodesign/Meridien
Mouse	*S*. Typhimurium monoclonal α-LPS C86309M	1∶200	1 h, 22°C	Meridien biosciences
Mouse	*Salmonella* spp. α-flagellin (I & II)	1∶800	2 h, 22°C	Biolegend & Maine Biotechnology Services
Rabbit	*Salmonella* Enteritidis O-Ag capsule (adsorbed polyclonal)	1∶100	Overnight, 4°C	Gift of Dr. Deanna Gibson [27],
Rabbit	αVi-ag antiserum	1∶400	2 h, 22°C	BD Difco
Rabbit	αCsgA	1∶400	2 h, 22°C	Gift of Dr. Matthew Chapman [22]
DAPI	DNA 10 uM	1∶1000	2 h with 2°ab	Sigma
Rabbit	*S*. Enteriditis, Typhimurium and Heidelberg “O” & “H”, cross reactive with *Enterobacteriaceae*	1∶200	2 h, 22°C	Novus Biologicals
